# The Value of Tumor Infiltrating Lymphocytes (TIL) for Predicting the Response to Neoadjuvant Chemotherapy (NAC) in Breast Cancer according to the Molecular Subtypes

**DOI:** 10.3390/biomedicines11113037

**Published:** 2023-11-13

**Authors:** Ionut Flaviu Faur, Amadeus Dobrescu, Adelina Ioana Clim, Paul Pasca, Catalin Prodan-Barbulescu, Bogdan Daniel Gherle, Cristi Tarta, Alexandru Isaic, Dan Brebu, Ciprian Duta, Bogdan Totolici, Gabriel Lazar

**Affiliations:** 1 IInd Surgery Clinic, Timisoara Emergency County Hospital, 300723 Timisoara, Romania; flaviu.faur@umft.ro (I.F.F.); drpascapaul@gmail.com (P.P.); catalin.proddan-barbulescu@umft.ro (C.P.-B.); tarta.cristi@umft.ro (C.T.); isaicus@gmail.com (A.I.); dr.brebudan@gmail.com (D.B.); ciprian_duta@yahoo.com (C.D.); 2 X Department of General Surgery, “Victor Babes” University of Medicine and Pharmacy Timisoara, 300041 Timisoara, Romania; 3 IInd Obstetric and Gynecology Clinic “Dominic Stanca”, 400124 Cluj-Napoca, Romania; clim.adelina@yahoo.com; 4 Department of Plastic, Reconstructive and Aesthetic Surgery, Rennes University Hospital Center, Université de Rennes, 16 Boulevard de Bulgarie, 35000 Rennes, France; gherlebogdandaniel@gmail.com; 5 Ist Clinic of General Surgery, Arad County Emergency Clinical Hospital, 310158 Arad, Romania; totolici_bogdan@yahoo.com; 6 Department of General Surgery, Faculty of Medicine, “Vasile Goldiș” Western University of Arad, 310025 Arad, Romania; 7 Department of Oncology Surgery, “Iuliu Hatieganu” University of Medicine and Pharmacy, 400347 Cluj-Napoca, Romania; dr.lazar.gabriel@gmail.com; 8 Ist Clinic of Oncological Surgery, Oncological Institute “Prof Dr I Chiricuta”, 400015 Cluj-Napoca, Romania

**Keywords:** breast cancer, TIL, modified radical mastectomy, breast conservative surgery, predictive biomarker

## Abstract

Introduction: The antitumor host immune response is an important factor in breast cancer, but its role is not fully established. The role of tumor infiltrating lymphocytes (TIL) as an immunological biomarker in breast cancer has been significantly explored in recent years. The number of patients treated with neoadjuvant chemotherapy (NAC) has increased and the identification of a biomarker to predict the probability of pCR (pathological complete response) is a high priority. Materials and methods: We evaluated 334 cases of BC treated with NAC followed by surgical resection from 2020–2022 at the Ist Clinic of Oncological Surgery, Oncological Institute “Prof Dr I Chiricuta” Cluj Napoca. Of the above, 122 cases were available for histological evaluation both in pre-NAC biopsy and post-NAC resection tissue. Evaluation of biopsy fragments and resection parts were performed using hematoxylin eosin (H&E). The TIL evaluation took place according to the recommendations of the International TIL Working Group (ITILWG). Results: There was a strong association between elevated levels of pre-NAC TIL. At the same time, there is a statistically significant correlation between stromal TIL and tumor grade, the number of lymph node metastases, the molecular subtype and the number of mitoses (*p* < 0.005). Intratumoral TIL showed a significant correlation with tumor size, distant metastasis, molecular subtype, number of mitosis, stage and lymph node metastasis (*p* < 0.005). We also demonstrated that high pre-NAC STIL represents a strong predictive marker for pCR. Conclusion: This study reveals the role of TIL as a predictive biomarker in breast cancer not only for the well-established TNBC (triple negative breast cancer) and HER2+ (Her2 overexpressed) subtypes but also in Luminal A and B molecular subtypes. In this scenario, the evaluation of sTIL as a novel predictive and therapy-predicting factor should become a routinely performed analysis that could guide clinicians when choosing the most appropriate therapy.

## 1. Introduction

The importance of the immune system as a potent anti-tumor defense has been consolidated in recent times, and novel immune-related therapies are, today, demonstrating a strong clinical benefit in the setting of several solid neoplasms. The composition of BC-infiltrating lymphocytes has been widely investigated so far; however, while it is commonly accepted that high TIL presence is associated with improved prognoses, the correlation between diverse TIL subpopulations and the responses observed experimentally is still characterized by a certain level of controversy [1]. Seventy-five percent of TILs are found to be T cells. Among these, CD8+ T cells represent the class of lymphocytes that correlate better with overall favorable clinical outcomes, usually infiltrating breast lesions in the largest proportion [2]. It has been widely documented that the presence of high rates of infiltrating CD8+ T cells is associated with overall longer survival rates [3]. NK cells serve as the first-line defense in association with CD8+ T lymphocytes. NKs are found to infiltrate breast lesions in a proportion ranging around 5% of the total lymphocytic population [4].

Tumor-infiltrating lymphocytes reflect the attempt of the host to eradicate malignancies, and have been explored during recent decades. Referring to the role of the immune system as a modulatory system within these tumor types, there are numerous controversies in the literature with the addition of numerous research studies [5,6]. The body’s antitumor response is based on the lymphocyte population (TILs–tumor infiltrating lymphocytes), an aspect intensively researched in the last decade [7]. Since an increasing number of breast cancer patients benefit from neoadjuvant therapy (NAC), it is crucial that we standardize a biomarker for predicting the tumor response rate [8,9,10,11,12,13,14]. The response rate to neoadjuvant therapy is standardized in four types: complete response (pCR), partial response (PR), stable pathology (SD), respectively, and progressive pathology in evolution (PD) [15,16,17,18]. TILs represent mononuclear cells belonging to the immune system, which can transition from the blood circulation level to the tumor level, where they initiate the immunomodulatory mechanism Although valuable information has been obtained, the heterogeneity in experimental design and TIL assessment has hindered a more comprehensive understanding of the biological value of TILs. The interaction of immune lymphocytes and tumor cells is cardinal in these procedures. In the immune system, lymphocytes can eradicate tumor cells and prevent neoplasm development through immune surveillance; tumor-infiltrating lymphocytes (TILs) participate in the regulation of the tumor niche and the inhibition of tumor formation and development [19]. This cell group presents a particular heterogeneity, being made up of cytotoxic T cells, T helper cells, B cells, macrophages, natural killer cells and dendritic cells, all these cell subspecies are part of the tumor microenvironment [20,21].

## 2. Materials and Methods

We evaluated 334 cases of BC treated with NAC followed by surgical resection from 2020–2022 at the Ist Clinic of Oncological Surgery, Oncological Institute “Prof Dr I Chiricuta” Cluj Napoca. Ethical clearance was obtained from the institutional ethics committee. Out of these, 122 cases were available for histological evaluation both in pre-NAC biopsy and post-NAC resection tissue. The surgical specimens were obtained from core needle biopsy and excisional biopsy of primary breast tumor. Four-micrometer-thick tissue sections from the surgical specimens fixed in 10% formalin and embedded in paraffin were reviewed, and representative tissue blocks were selected. There are two types of TILs: stromal and intratumoral. Stromal TILs (sTILs) are dispersed in the stroma and have no direct contact with carcinoma cells, while intratumoral TILs (iTILs) are defined as lymphocytes in direct contact with tumor cells. Evaluation of biopsy fragments and resection parts was performed using hematoxylin eosin (H&E). The TIL evaluation took place according to the recommendations of the International TIL Working Group (ITILWG) [22].

## 3. Histochemistry Analysis

The evaluation of the sTILs was performed according to the guidelines of the “International Working Group for sTIL in Breast Cancer—2014”. In detail, a section of 4–5 µm at a magnification of 200–400× was evaluated for each patient. The evaluation of the sTIL was performed using a percentage count of the stromal areas occupied by the lymphocyte and plasma cellular infiltrate, instead excluding the areas occupied by tumor cells. This evaluation considered only the mononuclear infiltrate within the borders of the invasive tumors. Large areas of central necrosis or fibrosis are not included in the evaluation. The stratification of the general study group by groups in relation to the sTIL value is distributed as follows (Table 6):

Group A—includes 17 patients with a value of sTIL between 0–10%
Group B—includes 41 patients with a value of sTIL between 10–40%
Group C—includes 62 patients with a value of sTIL between 40–90%
Group D—includes two patients with a value of sTIL over 90%

We evaluated 334 cases of BC treated with NAC (the majority of NAC regimens contained anthracycline and taxane.) Trastuzumab or lapatinib were typically used in HER2-positive patients, and followed by surgical resection from 2020–2022 at the Ist Clinic of Oncological Surgery, Oncological Institute “Prof Dr I Chiricuta” Cluj Napoca. Ethical clearance was obtained from the institutional ethics committee. Of the above, 122 cases were available for histological evaluation both in pre-NAC biopsy and post-NAC resection tissue. There are two types of TILs: stromal and intratumoral. Stromal TILs are dispersed in the stroma and have no direct contact with carcinoma cells, while intratumoral TILs are defined as lymphocytes in direct contact with tumor cells. Evaluation of biopsy fragments and resection parts was conducted using hematoxylin eosin (H&E). The percentage of stromal as well as intratumoral TILs was evaluated separately. iTIL was defined as the percentage of mononuclear cells within the epithelium of the invasive tumor cell nests. Stromal TIL (sTIL) was defined as the percentage of tumor stroma area that contains a lymphocytic infiltrate without direct contact to tumor cells. TIL assessment in the residual disease setting should be performed within the borders of the residual tumor bed, as defined by the presence of the residual tumor cells, in analogy with the definition of the residual tumor bed from the Residual Cancer Burden (RCB)-index [21]. The entire cross-sectional area of the residual tumor bed should be used for histologic TIL assessment. One section (4–5 μm) per patient can be considered to be sufficient for practical purposes. However, if the residual tumor bed is larger than 2 cm, more slides need to be assessed, with one slide for each cm of tumor bed as a minimum. For example, if the largest diameter is >5 cm, then at least five representative slides from the largest cross-sectional area should be considered. If the residual tumor bed is only 2 cm, one slide is considered enough. Thus, assessing numerous slides for each case should be possible mentioning the number of assessed slides specifically in the study protocol [19].

## 4. Statistical Considerations

Statistical analysis was performed using SPSS 23.0 for windows (SPSS, Inc., Chicago, IL, USA) which was used for data analysis. The associations between sTILs, iTILs and clinicopathological variables were examined using χ^2^ tests. Multivariable analysis of pCR was carried out using a binary logistic regression model. Normally distributed continuous data were expressed as means (SD) and were assessed using the analysis of variance (ANOVA), independent-sample *t*-test or paired *t*-test. Nonparametric data were analyzed using the Mann–Whitney and Wilcoxon tests. Two-sided tests were performed to declare statistical significance at *p* < 0.05.

## 5. Ethical Consent

All processes approached during the study with the inclusion of human subjects benefited from the approval of the ethics commission according to national and international standards in direct relation to the Helsinki declaration of 1964. This article does not include studies on laboratory animals. The consent mentioned above was received from, and approved by, each participant in the study (The Ethics Commission for Research and Development Activities and for Quality Assurance of Clinical Trials of the “Prof Dr Ion Chiricuță” Oncological Institute in Cluj Napoca, appointed by decision of the manager (IOCN no. 189-03.06.2021-Application no. 10442).

## 6. Results

The stratification of the study group was decided according to the molecular subtype based on the St Gallen classification. In accordance with this, 37.7% of cases (*n* = 46 cases) were included in Luminal A, followed by TNBC (triple negative breast cancer) in 25.9% (*n* = 31) of cases, respectively. Her2 was overexpressed in 20.49% (*n* = 25). A more limited number of cases were included in the Luminal B group (Her2- approximately 9.01%, Her2+ 7.37%, respectively) (Table 1).

Referring to the types of interventions performed within the study group, it can be observed that a significant percentage, 48.36% (59 cases), benefited from breast conservative surgery (BCS), and 22.95% (28 cases) from oncoplastic breast conservative surgery (OBCS), a fact that reinforces the idea of modulating the surgical therapeutic strategy depending on the tumor molecular subtype and the response rate to neoadjuvant therapy. A relatively small percentage, of approximately 28.66% (35 cases), benefited from a Madden–Auchincloss Modified Radical Mastectomy (MRM Madden–Auchincloss procedure). Current modern trends regarding oncological surgery of the mammary gland are related to the adoption of oncoplastic procedures, conservative procedures of the mammary gland without compromising oncological principles (Table 2).

The modulation of the surgical therapeutic strategy according to the molecular parameters and the response rate to the neoadjuvant therapy is a desideratum that should not be missing from the logistics of the medical–surgical team. The evaluation of some molecular, cellular and general parameters that can represent primary prognostic factors regarding the rate of therapeutic response, and at the same time the establishment of surgical management, is a leading topic worldwide.

From the point of view of the tumor topography, the majority of tumors (62.29%) were located at the ESQ level, respectively, and Spence’s axillary extension, followed by the ELQ (17.21%). A relatively small number of cases presented tumors located at the ISQ, ILQ and CQ level (approximately 20%). The impact of tumor topography on surgical procedures is a major one; favorable aesthetic results are defined by tumor location. A total of 60.65% of the diagnosed tumors were located in the left breast and 39.35% in the right breast, respectively (Table 3).

A microscopic analysis of the resection pieces shows the quantity and quality of cells at the level of the tumor bed, thus, a percentage of 63.11% presented a moderate threshold of cellularity at the level of the tumor bed (between 30–60%) and 15.57% presented a high degree of cellularity at the level of the tumor bed (over 60%), respectively. A percentage of 21.31% of all cases presented a low degree of cellularity at the level of the tumor bed. Rajan R. et al. published a study in 2004 that aimed to analyze the cellularity of the tumor bed after NAC. It highlights a significant decrease in cellularity in the context of neoadjuvant chemotherapy. In 2023, Damiano Gentile et al. published a study, including 495 patients, which analyzes the tumor response after NAC and the cellularity of the tumor bed as a prognostic factor in the case of breast cancer patients. This study concretizes the fact that a residual tumor-bed cellularity (RTC) value below 40% is associated with a longer disease-free interval (DFS) and an improvement in long-term survival, respectively. Another study published by Ahn S. et al. concludes that there is no statistically significant correlation between cellularity in the post-NAC tumor bed (RTC) and long-term survival. Referring to the characteristics of the study group, we can see that 58.19% (71 cases) of the cases are younger than 50 years old and 41.8% (51 cases) are older than 50, respectively. Regarding the hormonal status, 59.01% (72 cases) are represented by fertile or reproductive age patients, or premenopausal, and approximately 40.99% (50 cases) are represented by women with a postmenopausal status, respectively.

Microscopic analysis of tumor resection pieces highlights a preponderance of tumors with a G2 grading (mBloom-Richardson classification) in 44.26% of cases and a G1 and G3 in approximately 27.04% and 28.68%, respectively. Analysis was performed at the level of the study group, and the rate of mitotic proliferation at the level of the tumor bed, resulting in a percentage of 50% of cases with mitotic proliferation over 11 at the level of the tumor bed, a fact that can highlight an aggressive tumor profile. From the point of view of the dimensions, 59.83% of tumors (73 cases) have tumor sizes between 2–5 cm, 26.22% (32 cases) have tumor sizes over 5 cm and 13.93% (17 cases) have tumor sizes below 2 cm.

Analyzing tumoral lymphovascular invasion shows that 66.39% (81 cases) do not present lymphovascular invasion, and 33.66% (41 cases) present with lymphovascular invasion, respectively. Regarding the presence of distant metastases, in 78.68% (96 cases) they were absent, and in 21.31% (26 cases) they were present, respectively (Table 4).

The analysis of the histological types within the study group is presented in the above table. A predominance of infiltrative ductal carcinoma can be observed with a percentage of 72.95% (89 cases), followed by lobular carcinoma in a proportion of 13.93% (17 cases). Histological types such as mucinous, medullary or metaplastic carcinoma were identified in a significantly lower number (Mucinous 3.27% vs. Medullary 7.37%, respectively; Metaplastic 2.45%). An important analysis within the study group was represented by the evaluation of tumor response to neoadjuvant therapy. The evaluation of tumor response to neoadjuvant therapy can be quantified considering several classifications (Therasse, Miller-Payne, Chevallier, Sataloff classification).

In our group, we adopted the Therasse classification, that is Miller–Payne, in order to assess the degree of tumor response to neoadjuvant therapy. Thus, 27.04% (33 cases) of the cases presented a complete pathological response (pCR) to neoadjuvant therapy (NAC) and 34.42% (42 cases) presented a partial response, respectively. 23 No response to NAC therapy (stable disease-SD) without the dimensional change of tumor formations was shown in 23.77% of cases (29 cases) and 14.75% (18 cases) show dimensional changes of post-NAC formations, respectively, with an increase of over 20% compared to the initial size (Table 4). The stratification of the general study group by groups in relation to the sTIL value is distributed as follows (Table 5): Group A—includes 17 patients with a sTIL value between 0–10%; Group B—includes 41 patients with a value of sTIL between 10–40%; Group C—includes 62 patients with a value of sTIL between 40–90%; Group D—includes two patients with a value of sTIL over 90%.

Analyzing the response rate after NAC, we highlighted the fact that approximately 61.46% of patients presented a favorable response rate after NAC, whether we are talking about pCR or PR. In order to highlight the predictive value of sTIL through statistical analysis, we established a cut-off value regarding the potential response to NAC superimposed on each molecular subtype. Thus, for patients included in the molecular framework of Luminal A, a cut-off value of sTIL above 20% has a predictive potential regarding the favorable response rate to NAC (*p* = 0.05). Regarding the patients included in Luminal B, a cut-off value of sTIL of over 20% shows a predictive potential in terms of the response rate, whether we are talking about pCR or PR (>30%) (*p* = 0.093); 95% CI, 0.89–0.92).

We obtained statistically significant correlations between sTIL and tumor grade (0.152 [0.091, 0.262]; z = 6.80; *p* < 0.05), tumor size (0.154 [0.085, 0.198]; z = 5.72; *p* < 0.05) and molecular subtype (0.134 [0.090, 0.264]; z = 4.80; *p* < 0.05). However, we did not find a statistically significant correlation between sTIL and distant metastases (0.64 [0.78, 0.122]; z = 5.44; *p* = 0.1931), nor between sTIL and TNM stage (0.111 [0.76, 0.101]; z = 2.80; *p* = 0.068). Statistically significant correlations were obtained between iTIL and tumor grade, tumor size, distant metastases, TNM and molecular subtype (0.167 [0.091, 0.262]; z = 5.90; *p* < 0.05).

As can be seen (Table 6), we obtained different mean values depending on the molecular subtype, thus patients with tumors belonging to the non-luminal molecular subtype (HER2+ and TNBC) having higher values of sTIL and iTIL compared to patients belonging to the molecular subtype luminal (Luminal A and B).

Regarding the two TIL subtypes (intratumoral and stromal), it can be observed that iTIL is in direct correlation with the tumor stage according to the TNM classification (*p* = 0.01), while in the case of sTIL we did not obtain a statistically significant coefficient. Comparing the values obtained, it can be stated that iTIL has a higher specificity in relation to the tumor stage compared to sTIL, which does not sublimate the predictive potential of sTIL.

## 7. Discussions

In recent years, immunogenic studies and targeted therapies have been found increasingly frequently in the oncological therapeutic arsenal. Breast cancer is the best-studied neoplastic subtype from a histopathological and immunohistochemical point of view; in therapeutic dynamics there are many possibilities for therapeutic titration based on major predictive factors [23,24,25,26,27,28].

There have been numerous studies on the potentially predictive histopathological and immunohistochemical parameters regarding the response rate to neoadjuvant therapy in breast cancer [29,30]. The presence of STILs represents the expression of the antitumor immune response, and this marker could represent a major predictive factor regarding the therapeutic response after NAC [31,32,33,34,35,36]. Analyzing the specialized literature, we note that there are two types of lymphocytic tumor infiltrates (TILs), one with stromal localization and one with intra-tumoral localization [37]. Referring to the predictive value of the two types of infiltrates regarding the therapeutic response to neoadjuvant therapy in the case of breast cancer, there are numerous controversies in the medical literature [38,39,40].

Most of the studies with an impact from a qualitative and quantitative point of view show favorable results in favor of sTIL as a predictive marker in tumor response to neoadjuvant therapy compared to iTIL [41,42,43,44]. In relation to the molecular subtype of the tumor, most studies of TIL as a predictive factor of response to NAC give us favorable results in the case of HER2+ and TNBC patients, and only a few gives us proactive results in the case of patients classified as Luminal A and B subtypes. In 2010, Denkert et al. [20] documented a linear correlation between high levels of TILs, especially T cells, and clinical/radiological responses to anthracycline-based neoadjuvant-chemotherapy (NAC) regimens. Since this pivotal study, the predictive role of TILs with regard to the success of NAC has been highlighted by a considerable number of studies, often focusing on HER2-positive and TNBC molecular subtypes. The correlation between CD8+ T lymphocytes and Tregs as a valid indicator of success of NAC has been recently reported in the TNBC setting. Specifically, the CD8+/FOXP3+ ratio represents a reliable parameter, as it considers the relationship between immunosurveillance and immunosuppression cell compartments within the tumor bed [45].

The West trial demonstrates the fact that high values of sTILs increases the tumor response rate to neoadjuvant therapy and are associated with better long-term survival in the case of HER2 overexpression and TNBC patients [9,16,46,47]. The results from the literature show a particular heterogeneity regarding the predictive value of TIL in relation to the molecular tumor subtype [38,48]. The results obtained by us are in accordance with a study published by Denkert et al. which demonstrates the predictive value of TIL in relation to pCR in the case of all molecular subtypes of breast cancer [49]. We have obtained a statistically significant correlation between stromal TIL and tumor grade, tumor size, the number of distant metastases and molecular subtype (*p* < 0.05). Intratumoral TIL showed a significant correlation with tumor grade, tumor size, distant metastasis, molecular subtype, stage and lymph node metastasis (*p* < 0.05). We also demonstrated that high pre-NAC sTIL represent a strong predictive marker for pCR. In 2022, Shiqi Li et al. published a systematic review that included 29 publications demonstrating that high levels of sTIL can predict the response rate to NAC in breast cancer patients with a HER2+ molecular profile (OR = 2.54 95% CI, 1.50–4.29), respectively, in the case of patients with a TNBC molecular profile (OR = 3.67, 95% CI, 1.93–6.97). Mukta Pujani et al. demonstrate the existence of a significant correlation between stromal TIL and tumor grade, lymph node metastasis, molecular subtype and mitosis. Intratumoral TIL showed a significant correlation with tumor size, mitosis, tumor grade, distant metastasis, stage and lymph node metastasis [50].

Angelico, G. et al. show, in a prospective analysis, the fact that there is a heterogeneity regarding the value of iTIL and sTIL in relation to the tumor stage. They demonstrate a statistically significant correlation between iTIL and sTIL values, respectively, in stage I and II according to the TNM classification [51].

In the case of patients with a positive hormonal profile, this systematic review does not obtain statistically significant results (OR = 1.68, 95% CI, 0.67–4.25). In this systematic review, in the case of HER2+ and TNBC patients, the cut-off value of sTIL was 20%, a value from which significant results were obtained regarding the tumor response rate to NAC therapy. The conclusion of another meta-analysis published by Zhao-hua Gao et al. in 2020, which included 33 profile studies (18,170 patients) underlines the predictive value of sTILs regarding the tumor response rate to NAC in the case of patients with a TNBC and HER2+ molecular profile [52]. High values of sTILs are associated with a degree of favorable response to the NAC and with a longer-term survival (OS). In 2020, Lin He et al. published a meta-analysis that included 22 clinical trials (15,676 patients) demonstrating that each 10% increase in sTIL improves the long-term survival (OS) of patients with a HER2+ molecular profile (pooled Hazard ratio (HR), 0.92; 95% CI, 0.89–0.95) and TNBC (pooled HR, 0.90; 95% CI, 0.89–0.92). At the same time, this meta-analysis demonstrates that high levels of sTILs are associated with a favorable response rate to NAC therapy (pCR) regardless of the molecular subtype of breast cancer [53]. An important remark of the meta-analysis, the neoplasias included in the HER2+ and TNBC molecular subtypes showed significantly higher values of sTILs compared to the Luminal A and Luminal B subtypes (pooled HR, 1.06; 95% CI, 0.99–1.13)

## 8. Conclusions

Following our study, we can conclude that the TIL (iTIL and sTIL) value has a predictive potential regarding the response rate of breast tumors to neoadjuvant therapy (NAC) regardless of the molecular subtype. Different tumor-response cut-off values were highlighted depending on the molecular subtype, highlighting the fact that non-luminal tumors (TNBC and HER2+) present higher average values compared to luminal tumors (Luminal A and Luminal B). We consider that the TIL (stromal and intratumoral) value represents a reliable biomarker for predicting the tumor response to NAC and requires routine investigation in the case of all patients with breast neoplasm, and at the same time, the establishment of cut-off values at the global level through the implementation of large-scale population studies.

## Figures and Tables

**Table 1:** Stratification of the study lot according to the St Gallen classification.

Parameters	*n*	%
Luminal A	46	37.7
Luminal B Her2−	11	9.01
Luminal B Her2+	9	7.37
Her2+	25	20.49
TNBC	31	25.9

**Table 2:** Distribution of the batch according to tumor location and type of surgical intervention.

Parameters	*n*	%
Unifocal	81	66.39
Multifocal	26	21.31
Multicentric	15	12.29
The type of intervention		
MRM	35	28.66
OBCS	28	22.95
BCS	59	48.36

**Table 3:** General clinical-paraclinical characteristics.

Parameters	*n*	%
**Age**		
<50	71	58.19
≥50	51	41.8
**Menopausal status**		
Premenopausal	72	59.01
Postmenopausal	50	40.99
**Grading (mBloom Richardson)**		
1	33	45,043
2	54	44.26
3	35	28.68
**Mitotic Count**		
0–5	25	20.49
45,205	36	29.50
≥11	61	50
**Tumor size**		
<2	17	13.93
45,048	73	59.83
>5	32	26.22
**Lymphovascular invasion**		
Present	41	33.66
Absent	81	66.39
**Distant metastases**		
Present	26	21.31
Absent	96	78.68
**TNM**		
I	17	13.93
II	73	59.83
III	24	19.67
IV	8	20,241
**Location**		
External superior quadrant (ESQ)	76	62.29
Internal superior quadrant (ISQ)	12	30,560
External lower quadrant (ELQ)	21	17.21
Internal lower quadrant (ILQ)	7	26,785
Central quadrant (CQ)	6	33,329
**Post-NAC tumor bed cellularity (RTC)**		
below 30%	26	21.31
30–60%	77	63.11
over 60%	19	15.57
**Breast**		
Right	48	39.35
Left	74	60.65

**Table 4:** Anatopathological characteristics of the batch.

Parameters	*n*	%
**NAC response**		
pCR ^i^	33	27.04
PR. ^ii^ (over 30%)	42	34.42
SD ^iii^	29	23.77
PD ^iv^ (over 20%)	18	14.75
**Histological type**		
Infiltrative Ductal Carcinoma	89	72.95
Lobular	17	13.93
Mucinous	4	3.27
Medullary	9	7.37
Metaplastic	3	2.45

^i^ pCR = complete response to NAC (neoadjuvant therapy—based on anthracyclines and taxanes +/− targeted therapy—Transtuzumab or Lapatinib); ^ii^ PR = partial response to NAC (dimensional reduction of the tumor over 30%); ^iii^ SD = no response to NAC, stable disease; ^iv^ PD = progressive disease below NAC with tumor size increase (over 20%).

**Table 5:** Stratification of the batch into groups according to the value of sTIL.

GROUP	No. of Cases	%
A	17	13.93
B	41	33.6
C	62	50.81
D	2	1.63

**Table 6:** Values of sTIL and iTIL in the context of various clinicopathological parameters.

Parameters	*n*	Stil (Mean ± SD)	*p* Value	ITIL	*p* Value
**T Grade**					
1	33	7.01 ± 3.9	0.001	21.71 ± 11.03	0.001
2	54	39.4 ± 19.06		73.01 ± 39.03	
3	35	68.63 ± 20.13		171.31 ± 41.32	
**Tumor Size**					
2	17	41.23 ± 22.13	0.002	83.41 ± 44.24	0.002
2–5	73	57.28 ± 29.17		101.38 ± 33.17	
≥5	32	69.11 ± 20.19		153.31 ± 42.19	
**Molecular Subtype**					
Luminal A	46	21.29 ± 17.55	0.001	43.12 ± 29.12	0.002
Luminal B	20	33.19 ± 19.23		68.47 ± 38.12	
HER2+	25	44.21 ± 28.24		98.42 ± 56.44	
TNBC	31	69.11 ± 27.31		128.12 ± 54.12	
Distant Metastasis					
Present	26	71.23 ± 23.97	0.1931	162.23 ± 46.32	0.005
Absent	96	44.23 ± 27.28		95.21 ± 61.12	
**Lymphovascular Invasion (Lvi)**					
Present	41	68.12 ± 28.67	0.001	152.21 ± 50.12	0.05
Absent	81	39.19 ± 27.12		79.12 ± 54.18	
**TNM**					
I	17	31.23 ± 21.83	0.068	62.46 ± 54.12	0.01
II	73	48.23 ± 28.13		91.46 ± 51.12	
III	24	68.23 ± 27.13		138.18 ± 46.23	
IV	8	61.17 ± 23.97		141.12 ± 44.23	

## Data Availability

Data are contained within the article.
